# Contrastive Learning for 3D Point Clouds Classification and Shape Completion

**DOI:** 10.3390/s21217392

**Published:** 2021-11-06

**Authors:** Danish Nazir, Muhammad Zeshan Afzal, Alain Pagani, Marcus Liwicki, Didier Stricker

**Affiliations:** 1 Department of Computer Science, Technical University of Kaiserslautern, 67663 Kaiserslautern, Germany; danish.nazir@dfki.de (D.N.); didier.stricker@dfki.de (D.S.); 2 Mindgarage, Technical University of Kaiserslautern, 67663 Kaiserslautern, Germany; 3 German Research Institute for Artificial Intelligence (DFKI), 67663 Kaiserslautern, Germany; alain.pagani@dfki.de; 4 Department of Computer Science, Luleå University of Technology, 971 87 Luleå, Sweden; marcus.liwicki@ltu.se

**Keywords:** point cloud classification, point cloud shape completion, AutoEncoders, contrastive AutoEncoders, contrasitive learning for point clouds, self-supervised learning for point cloud shape completion

## Abstract

In this paper, we present the idea of Self Supervised learning on the shape completion and classification of point clouds. Most 3D shape completion pipelines utilize AutoEncoders to extract features from point clouds used in downstream tasks such as classification, segmentation, detection, and other related applications. Our idea is to add contrastive learning into AutoEncoders to encourage global feature learning of the point cloud classes. It is performed by optimizing triplet loss. Furthermore, local feature representations learning of point cloud is performed by adding the Chamfer distance function. To evaluate the performance of our approach, we utilize the PointNet classifier. We also extend the number of classes for evaluation from 4 to 10 to show the generalization ability of the learned features. Based on our results, embeddings generated from the contrastive AutoEncoder enhances shape completion and classification performance from 84.2% to 84.9% of point clouds achieving the state-of-the-art results with 10 classes.

## 1. Introduction

Real-world 3D data is the output of various 3D sensors such as LiDAR scanners and RGB-D cameras. The raw output of these sensors, especially LiDAR scanners, contains a large amount of missing data. Furthermore, it is also unstructured, which means that the distance between neighboring points is not always fixed [1]. Therefore, it is challenging to apply conventional deep learning methods directly on the raw outputs [1,2]. For instance, convolutional neural networks cannot be applied directly on point clouds to learn feature representations because Convolution operation requires the data to follow an order and exist on a structured grid [1,2].

Recently, graph-based convolutional neural network (GCNNs) methods have been very successful in learning point cloud representations tasks [3,4,5]. Several state-of-the-art methodologies such as PointNet [3], PointNet++ [5], and dynamic graph convolutional neural networks (DGCNN) [4] are introduced, which aims to recover the topology of the point clouds by learning-rich representations. GCNNs based methodologies make use of deep AutoEncoders, which have millions of learnable parameters, making them vulnerable to over-fitting [6]. Large-scale annotated datasets are required for training GCNNs to have a generalizable solution for shape completion and classification problem. However, creating labeled ground truth data for point clouds is challenging, and expensive [7]. Therefore, unsupervised learning methods, also known as self-supervised learning methods that can learn from unlabeled data, have drawn significant attention [7].

**Figure 1:**
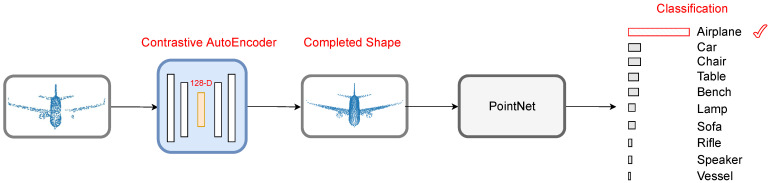
The incomplete shapes are passed through a contrastive AutoEncoder, which completes the shape. The completed shape is sent to the pre-trained PointNet [3] for classification.

**Figure 2:**
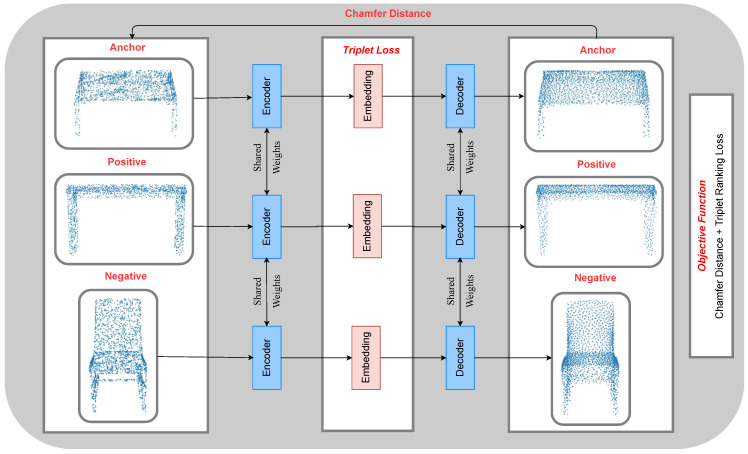
Forward pass of the proposed contrastive AutoEncoder. The triplets are passed through the encoder to transform high-dimensional point clouds to lower-dimensional feature representations of the point clouds. Triplet loss between anchor, positive, and negative feature representations is calculated, and they are sent to the decoder, which reconstructs the point clouds. Finally, the reconstruction loss is calculated between the decoded and input point clouds. The objective function is formulated as the sum of these two losses. The network is trained in an end-to-end manner.

Self-supervised learning (SSL) consists of pretext and downstream tasks. In our work, the pretext task consists of learning point cloud representations. Downstream tasks utilize the learned representations by pretext task. We choose point cloud classification as a downstream task and use pre-trained PointNet [3] to measure the classification accuracy. The proposed contrastive AutoEncoder is optimized over contrastive loss [8,9,10,11,12,13] and a distance function. Contrastive learning is a variant of self-supervised learning, and we use it as our pretext task. The intuition behind contrastive learning is that objects belonging to the same class should be closer in feature space than objects of a different class. Our network focuses on learning both global and local feature representations of the point clouds. The global feature representations of the classes are learned through contrastive loss, whereas the local feature representations are learned through a distance function. Figure 1 shows the complete workflow of our approach. The incomplete shapes are generated by removing 20% points uniformly at random of ShapeNet core [14] dataset. The proposed contrastive AutoEncoder completes the shapes, and in the last stage, PointNet classifies the completed shape. The forward pass of the proposed network is given in Figure 2.

Our key contributions are as follows:

- We train an end-to-end deep contrastive AutoEncoder that does not rely on intermediate feature computations such as embedding;
- The combination of local and global features has not been investigated in the context of point cloud shape completion and classification. Global features are utilized in [6,15,16] methods as a pretext to task learning, whereas local features are explored in [17,18] approaches. We combine local and global feature learning into a single task and apply it to classification and shape completion problems.

## 2. Related Works

Point cloud processing using machine learning techniques has obtained increased attention over the past few years. We will review related point cloud analysis and shape completion methods.

### 2.1. Point Cloud Analysis

There exist two subcategories for point cloud analysis methods. The first category is supervised feature learning, which employs full ground truth for learning feature representations. The second category is unsupervised learning methods, which only needs partial ground truth data to learn features representation from point clouds. The following sections will discuss all of the previous approaches to shape completion and classification.

#### 2.1.1. Supervised Learning Methods

Supervised learning methods require labeled data of point clouds. The types of methods which are used in this domain are given below.

Traditional Methods: Traditional methods for learning point cloud representations include hand-crafted-based techniques. The aim of traditional techniques is to capture the local geometric structure information of point clouds. By utilizing the local geometric information, intrinsic [19,20] or extrinsic feature descriptors [21,22] can be created. Extrinsic descriptors use the coordinates of the shapes in 3D space, whereas intrinsic descriptors discretize the 3D shape as a mesh or a graph. Such feature descriptors are very limited and include manual work.

Convolutional Methods: The idea of convolutions can also be extended to 3D from 2D convolutions and used for feature extraction. Convolution-based methods have better performance on downstream tasks than traditional handcrafted features. There are various ideas to use CNN with point clouds. One way is to perform voxelization of 3D point clouds, which means that we create a voxel structure of [X×Y×Z] and then we can perform convolution operation using filters of size [x×y×z] with [x,y,z]≤[X,Y,Z] [23,24,25,26]. However, these methods have a quantization effect and have high computational costs. MultiView methods [27,28,29] is another way of applying CNN’s to 3D data. The idea behind MultiView methods is to convert 3D data to 2D and then apply existing CNN techniques. The accuracy of MultiView methods is greater than voxel-based methods because they make use of existing state-of-the-art 2D techniques and extend them to 3D data.

Graph-based Methods: The idea behind graph-based methods is to capture the local structure of the point clouds. They represent each point in the point cloud by a node, allowing us to model correlations within the point clouds. Deep graph-based convolutional networks (DGCNN) [4] propose a dynamic graph edge convolution (EdgeConv) on point clouds. EdgeConv focuses on exploiting the geometric structure on point clouds. It constructs a local neighborhood graph for each point based on the distances of point features to its neighbors. It then applies the convolution operation on edge in metric space, which connects the neighborhood pair of the points. Recently, edge-aware point cloud completion [30] has extended the idea of EdgeConv. It uses an edge-aware feature expansion (EFE) module to upsample point features in their proposed deep AutoEncoder. The EFE module features help preserve the local edges, which are important point cloud descriptors.

Learning from raw 3D point clouds: In other methods, e.g., in convolutional-based methods, we first had to calculate intermediate representation such as voxels, 2D grids or graphs, and then learn features. However, instead of computing intermediate representation, we can learn features directly from unstructured raw point clouds.

PointNet [3] is the first method to process unstructured point clouds directly. It individually processes each point in 3D data, and therefore disarrangement in the point clouds will not affect the model. However, as a consequence, PointNet will not be able to utilize the local structure of the 3D point cloud. To counter this, PointNet++ [5] was introduced later, which applied PointNet recursively on a nested partitioning of the input point set. TopNet [17] expanded further on the idea of local structure learning from point clouds. It proposes a rooted tree architecture in the decoder for feature extraction without assuming any specific topology in the point cloud structures.

#### 2.1.2. Unsupervised Learning Methods

Labeled 3D point cloud data is often hard to obtain for new applications. This fact allowed the boom of many Unsupervised learning methods, which learn features from unlabeled data to solve this problem. One of the solutions to this problem is to introduce pretext task learning [6,15,16,31]. In pretext task learning, the goal is to learn global features by performing a mainstream task. Initial approaches considered orientation estimation prediction [15] and reconstruction [16] of point clouds as the pretext task. In orientation estimation, we rotate point clouds and train the classifier to predict the rotation angle, whereas, in reconstruction, we learn by applying reconstructions of generated deformed point cloud shapes. Recently, SSL by learning discrete generative models [31] proposed a pretext task where the aim is to learn data distribution through generative models.

Recently, fully convolutional geometric features (FCGF) [32] and contrast-Net [6] was introduced, which uses the concept of contrastive learning. In FCGF [32], 3D CNN with fully-convolutional-geometric-layers are used, and it is trained using triplet loss. Contrast-net [6] uses DGCNN for feature extraction and apply pairwise ranking loss. It gives positive and negative pairs to DGCNN and applies pairwise ranking loss. Positive pairs are generated by selecting samples randomly and ensuring that they are from the same class, whereas negative pairs are generated using the same technique, ensuring that positive and negative pairs represent different classes. The learned features in the classification downstream task. LatentGANs [33] introduced a new deep auto-encoder network with state-of-the-art reconstruction quality and generalization ability for point cloud data. Similarly, FoldingNet [34] is also an end-to-end AutoEncoder. It introduced a Graph-based enhancement applied to the encoder to enforce local structures on top of PointNet, and a folding-based decoder deforms a canonical 2D grid onto the underlying 3D object surface of a point cloud.

### 2.2. Shape Completion

3D point cloud shape completion is a challenging problem in computer vision. The applications of shape completion range from robotics to autonomous vehicles, and there has been significant development of methods in the field [35,36,37,38,39,40]. Earlier approaches [35,36,38] to shape completion were inspired by the performance of 2D CNN operations on images, and they extended the idea and introduced 3D convolutions on voxels. They have produced promising reconstruction results, however, voxels data representation have memory constraints and cannot produce high-resolution outputs. PCN [40] was one of the first approaches which discarded the voxel format and incorporated mesh formats. Recent works, Top-Net [17], PMP-Net [18], and RLGAN [39] have gradually discarded the voxel format, and now they are moving towards meshes, i.e., they use a mesh format to represent the 3D point cloud shapes. Mesh representation allows the consumption of point clouds directly into the network without dealing with any intermediate representation. It provides a significant advantage over voxel representation. Therefore, we also use the meshes format to represent our point cloud shapes.

As discussed above, the earlier approaches focus mainly either on global feature learning or on local feature learning by utilizing pretext tasks. However, we propose a unique combination of global and local features that learns local topology and considers global features of the respective classes.

## 3. Methods

We used AutoEncoder [41] as our backbone network to process unstructured point clouds. We added more convolutional layers into the backbone network and changed the objective function to adapt to our problem. It helped us achieve much stable and efficient training, and it also helped us get better reconstruction quality.

### 3.1. AutoEncoder

**Figure 3:**
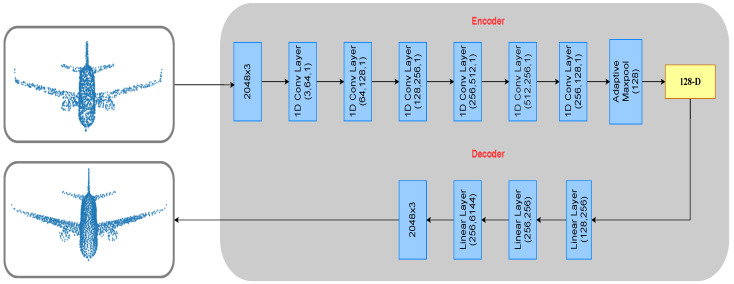
The encoder consists of 1D convolutional layers, which are applied until we get 128−D embedding. Batch normalization of the input channel size is applied to each layer. The decoder consists of only fully connected layers and maps the embedding to full point cloud of shape (2048×3 ).

AutoEncoder (AE) is an unsupervised method in which we learn feature representation of the data through two networks, i.e., encoder and decoder. The encoder converts the complex input into encoded representation, whereas the decoder reverts the encoded version to the original dimension. The loss is calculated between the output of the decoder and input. However, to extract global representations, we also introduced contrastive loss at the encoded representations of the data. Figure 3 shows the AE architecture of our method. The encoder produces the intermediate feature representation of the incomplete point cloud, and the decoder uses the produced intermediate representation to generate a dense point cloud. The following sections give more details about the objective function of our AutoEncoder.

#### 3.1.1. Objective Function

We use a combination of a distance function and contrastive loss to train our network. The proposed method extracts local features of the point clouds from the distance function, whereas it extracts global features using contrastive loss.

Distance Function: Distance functions that are suitable to our problem are Earth Mover’s distance (EMD) [42] and Chamfer’s distance [43]. Earth Mover’s distance is harder to optimize, and in practice, it gives only an approximate solution [43]. Therefore, we chose Chamfer distance over Earth Mover’s distance [39,43]. Furthermore, we employ symmetric Chamfer distance to measure the quality between the input and decoder’s output. It is defined as follows.

(1) $$\begin{array}{c} L\left(P_{1},P_{2}\right)=\sum_{a\in P_{1}}min_{b\in P_{2}}||a-{b||}_{2}^{2}\\ +\sum_{b\in P_{2}}min_{a\in P_{1}}||a-{b||}_{2}^{2} \end{array}$$

where $P_{1}\in R^{2048\times 3}$ denotes the Input point cloud from the training set and $P_{2}\in R^{2048\times 3}$ denotes the encoder’s generated point cloud representation, which is decoded using the decoder. In the first term, the subscripts *a* and *b* represent the 3D points $P_{1}$ and $P_{2}$, whereas, in the second term, they represent 3D points in $P_{2}$ and $P_{1}$.

Contrastive Loss: There are two main ideas for loss functions in contrastive learning space on which we can train our network.

The first idea is pairwise ranking loss, which is also used in related work [6]. It uses use pairs of positive and negative training data points. Positive pairs are formulated by choosing an anchor sample and a positive sample similar to an anchor. Negative pairs consist of an anchor sample and a negative sample dissimilar to an anchor. The objective is to learn representations with a smaller distance between them for positive pairs and greater distance than some margin values for negative pairs.

The second idea is triplet ranking loss [8,9,10,11,12,13] which uses the concept of triplets. A triplet is formed by an anchor sample, a positive sample, and a negative sample. A positive sample means that it should be similar to an anchor, whereas a negative sample means the opposite. This setup outperforms pairwise ranking loss, and also, it is easier to optimize on. Hence we use triplet ranking loss in our approach.

(2) $$\begin{array}{c} d\left(r_{a},r_{p}\right)=\sum_{i\in r_{a},j\in r_{p}}\left|\right|r_{a}^{i}-r_{p}^{i}{\left|\right|}_{2}^{2} \end{array}$$

(3) $$\begin{array}{c} d\left(r_{a},r_{n}\right)=\sum_{i\in r_{a},j\in r_{n}}\left|\right|r_{a}^{i}-r_{n}^{i}{\left|\right|}_{2}^{2} \end{array}$$

We use the $l_{2}$ distance metric to find the distance between the anchor, positive, and negative samples. Equation (2) represents the distance between representations of anchor and positive sample and Equation (3) represents the distance between representations of the anchor and negative sample.

Using Equations (2) and (3), triplet ranking loss can be defined as follows.

(4) $$\begin{array}{c} L\left(r_{a},r_{p},r_{n}\right)=max\;\left(0\;,\;m\;+\;d\left(r_{a},r_{p}\right)\;-\;d\left(r_{a},r_{n}\right)\right) \end{array}$$

where $r_{a}\in R^{2048\times 3}$ denote anchor sample, $r_{p}\in R^{2048\times 3}$ denote positive sample, $r_{n}\in R^{2048\times 3}$ denote negative sample, and $m\in R^{d}$ denotes minimum margin between positive and negative samples.

There arise three different scenarios from Equation (4), which are discussed as follows.

- $d\left(r_{a},r_{n}\right)>d\left(r_{a},r_{p}\right)+m$: This means that negative samples are already sufficiently distant to anchor samples in the embedding space, which will in turn cause loss to 0 and the network will not learn anything. This kind of triplets are known as easy triplets;
- $d\left(r_{a},r_{p}\right)>d\left(r_{a},r_{n}\right)$: Negative samples are closer to the anchor than positive samples and this will cause a loss to be positive and have a greater margin than *m*. The network will then learn something and such triplets are known as hard triplets.
- $d\left(r_{a},r_{p}\right)<d\left(r_{a},r_{n}\right)<d\left(r_{a},r_{p}\right)+m$: Negative samples are more distinct to the anchor but the distance is not greater than margin *m*. Hence, the loss is still positive, encouraging the network to learn something, but less than *m*. They are known as semi-hard triplets

Proposed Loss: The proposed objective of our methodology is given as follows.

(5) $$\begin{array}{c} L\left(r_{a},r_{p},r_{n}\right)=L_{triplet}\left(r_{a},r_{p},r_{n}\right)+L_{ch}\left(r_{a},dec\left(e_{a}\right)\right)\\ +L_{ch}\left(r_{p},dec\left(e_{p}\right)\right)+L_{ch}\left(r_{n},dec\left(e_{n}\right)\right) \end{array}$$

where $L_{ch}$ and $L_{triplet}$ represents the terms defined in Equations (1) and (4). The embeddings generated using the encoder for the anchor, positive, and negative samples are represented by $e_{a}$,$e_{p}$ and $e_{n}$$\in R^{128}$. The embeddings are sent to the decoder, which is represented as dec to get a full point cloud $R^{2048\times 3}$.

## 4. Experiments and Results

 We conduct extensive experiments to prove the usefulness of the learned features through our approach. To observe the effect of the combination of local and global features on shape completion and classification, we also provide the results where the network is optimized only on local features. This network is termed as a naive AutoEncoder and has the same architecture as a contrastive AutoEncoder. However, it is trained only with the Chamfer distance function. This experiment will prove our claim that contrastive learning helps in classification and shape completion pipelines.

In the first part of our experiments, we choose four classes that are the same as used by previous work [39]. In the second part, we extend the number of classes from 4 to 10 and train previous work [39] for a fair comparison. After training, we evaluate shape completion by using the mean Chamfer distance to the ground truth. The evaluation of completed shapes classification is done by computing classification accuracy using PointNet.

### 4.1. Implementation Details

We used PyTorch [44] for the implementation and training of our network. In the forward pass of the network, It calculates triplet ranking loss and Chamfer distance, and then back-propagation is performed on the sum of these two losses as shown in Equation (5). The complete training steps are given in Algorithm 1.
**Algorithm 1** Training Contrasitive AutoEncoder.1:**for** epoch=1,…total_epochs **do**2:    **for** i,datainenumerate(train_loader) **do**3:   Extract online triplets and ground truth.4:   $r_{a}$, $r_{p}$, $r_{n}$ = $train\_loader[“triplet^{”}]$5:   $e_{a}$, $e_{p}$, $e_{n}$ = $Encoder\;(r_{a},r_{p},r_{n})$6:   $dec\_e_{a}$, $dec\_e_{p}$, $dec\_e_{n}$ = $Decoder\;(e_{a},e_{p},e_{n})$7:   Compute Chamfer Loss and triplet loss.8:   triplet_loss = $compute\_triplet\_loss\;(e_{a},e_{p},e_{n})$9:   anchor_ch = $compute\_chamfer\;(r\_a,dec\_e_{a})$10:   pos_ch = $compute\_chamfer\;(r\_p,dec\_e_{p})$11:   negative_ch = $compute\_chamfer\;(r\_n,dec\_e_{n})$12:   Calculate and backpropogate the total loss.13:   chamfer_losses = positive_ch + negative_ch + anchor_ch14:   total_loss = triplet_loss + chamfer_losses15:   total_loss.backward()16:    **end for**17:**end for**

Computing the Chamfer distance is straightforward. However, triplet ranking loss requires careful design decision for mining triples. Triplet mining refers to the method of extracting triplets from the dataset. Each triplet contains one sample from anchor, positive and negative class. Triplet mining for the triplet ranking loss is an essential factor, and chosen strategy will have a high impact on training efficiency and final performance. There exist 2 strategies for choosing triplets which are explained below.

- Offline triplet mining: The traditional way of defining triplets is to define them either at the beginning of the training or after every epoch. It is done by computing all of the training set embeddings and select hard or semi-hard triplets.
- Online triplet mining: The idea of online triplet mining requires defining triplets for every batch of inputs during training. It does not requires defining the triplets before the training.

Offline triplet mining requires performing forward pass through all of the training data before training. It also requires updating the triplets after each epoch of training. Therefore, it can be very time-consuming and inefficient. However, online triplets mining does not require pre-computation since it produces triplets for each batch of input. It also provides better training efficiency and avoids easy triplets [9]. Therefore, we chose online triplet mining to train our network.

#### Hyper-Parameters and Optimization

We use the Adam optimizer and a learning rate of 0.001 and weight decay of 0.001, which helps us stabilize the contrastive loss. We also use a learning rate scheduler to further reduce the learning rate after [100,175,250,400,800] epochs. The optimal value for the margin *m* of triplet ranking loss for our data-set is 0.5, and we use $l_{2}$ norm as our distance measure between anchor, positive, and negative representations.

### 4.2. Dataset

The ShapeNet [14] dataset consists of 3D model shapes, which like famous Image-Net [45] dataset, are categorized according to WordNet [46] noun synsets. The raw shapes in the ShapeNet dataset come from different existing research and open-source 3D repositories [14]. The geometric annotations of the raw shapes, including shape alignment, symmetry, and object size are refined by algorithmic predictions, and manual annotations [14]. We use ShapeNetCore [14,33] dataset, which is a subset of ShapeNet dataset [14] to perform all of our experiments. It consists of single 3D model shapes that are semantically distinct objects for benchmarking. It contains 55,000 unique 3D model shapes with a point density of 2048×3 and covers up to 55 common object categories. We chose two samples of the ShapeNetCore dataset [14,33], which consists of 4 and 10 classes based on having the most number of shapes. We chose the 4 classes: Airplane, Chair, Table, and Car and extended this set and added 6 more classes, Bench, Lamp, Speaker, Rifle, Sofa, and Vessel, to make 10 classes dataset. Due to a high imbalance in the dataset, we replicate samples so that all the classes have approximately the same number of samples. The total number of shapes for the 4 classes dataset sums up to 22,803, and for 10 classes, it sums up to 70,000 samples in the dataset. The shape of our dataset for 4 and 10 classes is (22,803, 2048, 3) and (70,000, 2048, 3), respectively.

T-distributed stochastic neighbor embedding (TSNE) [47,48,49] is a method of nonlinear dimensionality reduction. It projects a high dimensional dataset into lower dimensions without losing much structural information. It helps to create 2D or 3D visualizations. We use TSNE to project our dataset and embeddings into 2D plots. In the case where we have four classes, TSNE transforms (22,803, 6144) dimensional data to (22,803, 2) whereas, in the case of 10 classes, it projects (70,000,6144) dimensional data to (70,000, 2). The visualizations are important because it helps to ensure that the dataset is not separable. It is important to know this fact because if the dataset is separable, it does not make any sense to apply contrastive learning to such a dataset.

**Figure 4:**
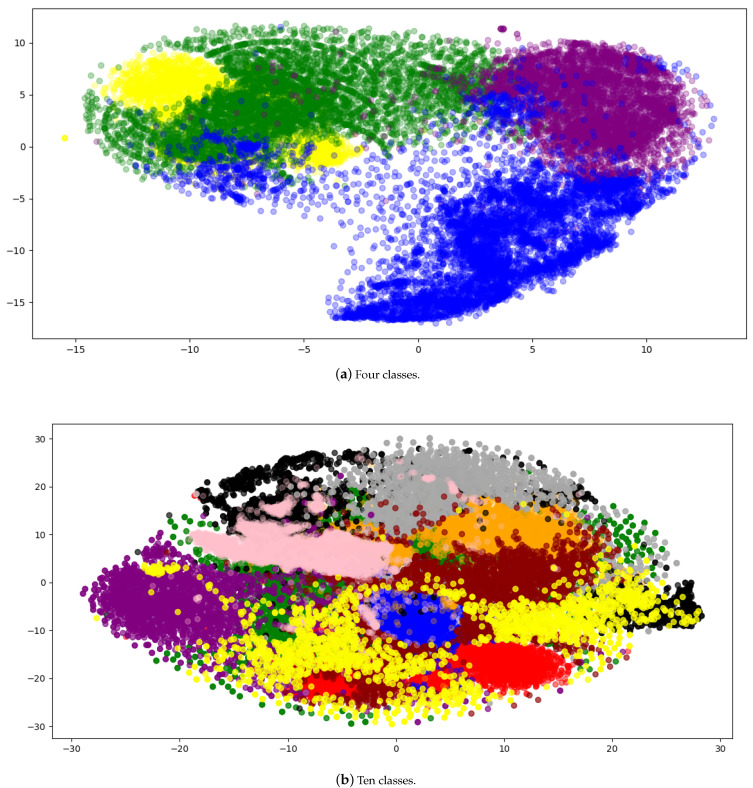
The visualizations are made by projecting the dataset into a two-dimensional space. Part (**a**) shows the dataset visualization of 4 classes whereas (**b**) shows the visualization of 10 classes.

Figure 4 shows the TSNE plots of 4 and 10 classes plots. Figure 4a shows that there is a somewhat clear separation between the classes. From the 10 classes plot given in Figure 4b, we can infer that contrastive learning can be applied to this dataset as the boundaries are not clearly defined for each class.

### 4.3. Embeddings

Embeddings produced by all of the methods we are using in this work are 128 dimensional for each shape. In the case where we have four classes, we transformed the embeddings from (22,803, 128) to (22,803, 2). On the other hand, for 10 classes we transformed (70,000, 6144) dimensional data to (70,000, 2). We use TSNE to apply the transformations mentioned above. It also allows us to compare the embeddings plot with the original dataset.

**Figure 5:**
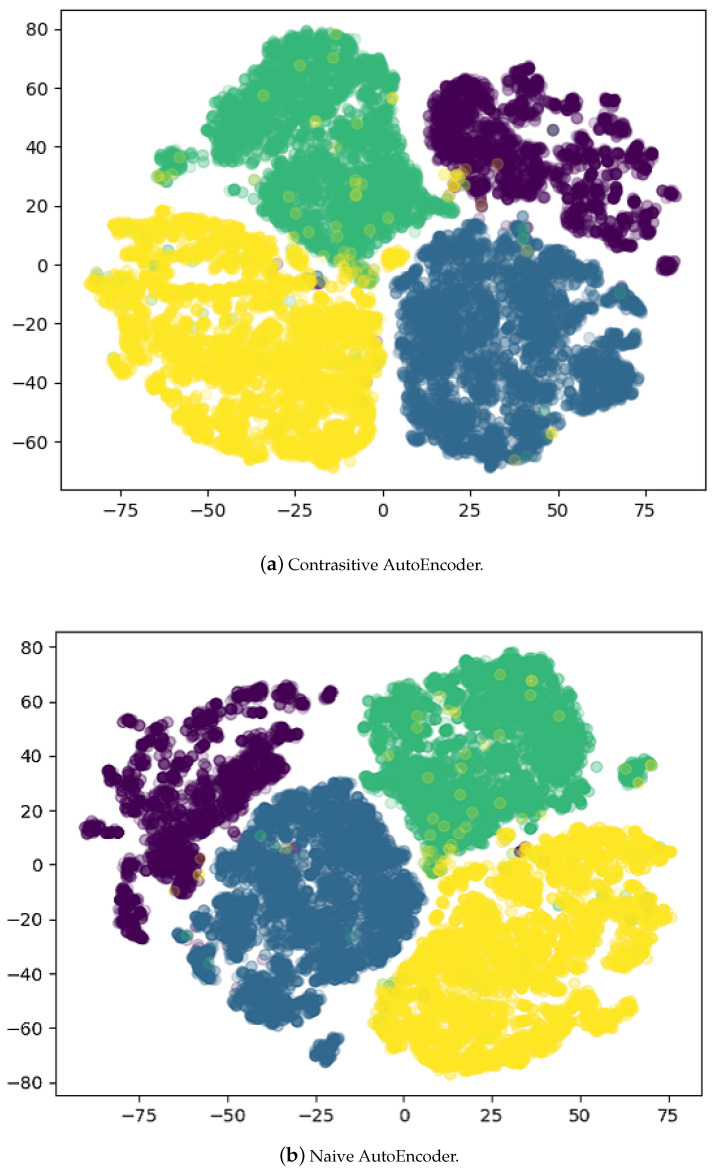
Embedding visualization of dataset with 4 classes. Part (**a**) shows the learned contrastive AutoEncoder i.e., with contrastive loss embeddings visualization whereas (**b**) shows the visualization of the naive AutoEncoder i.e., without contrastive loss. Contrastive AutoEncoder embeddings are similar to the naive AutoEncoder.

**Figure 6:**
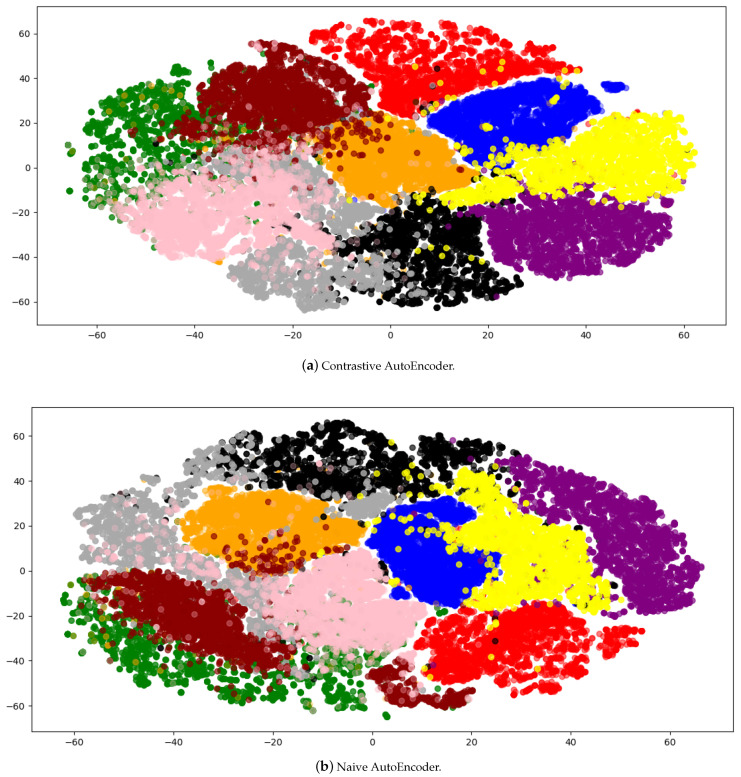
Embedding visualization of the dataset with 10 classes. Part (**a**) shows the learned contrastive AutoEncoder, i.e., with contrastive loss embeddings visualization whereas (**b**) shows the visualization of the AutoEncoder without contrastive loss.

Figure 5a and Figure 6a shows the embeddings generated by the contrastive AutoEncoder, and the boundaries for each class are very well defined except for two classes. We investigated this behavior and found that the shapes for these classes are similar and challenging to distinguish, even for human observers. On the other hand, Figure 5b and Figure 6b represent the embeddings learned by the AutoEncoder trained only on Chamfer distance.

### 4.4. Shape Completion

**Figure 7:**
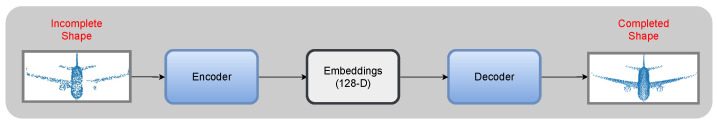
Shape completion pipeline. The incomplete shapes with 1638×3 are passed through the contrastive AutoEncoder to receive complete 2048×3 shape.

We generated test data shapes with 20% missing points to test our AutoEncoder shape completion capabilities. It is done by selecting a random point from the incomplete point cloud and removing its neighboring points within a certain radius. Furthermore, the value of the radius varies for different classes. After the shapes with missing data are generated, they are passed from the shape completion pipeline to generate complete shapes, as shown in Figure 7. Table 1 shows the quantitative results, i.e., the mean Chamfer distance per point for completed shapes.

### 4.5. Classification

After completing shapes using the RLGAN, naive AutoEncoder, and contrastive AutoEncoder, we also measure classification accuracy using PointNet. The completed shapes are passed to a pre-trained PointNet, which outputs the classes for each completed shape. Table 2 depicts the classification accuracy of the methods mentioned above.

When the number of classes is four, the naive AutoEncoder and contrastive AutoEncoder almost produce the same classification accuracy. The shapes used in the four classes are semantically very different, making them easier to classify, and using contrastive learning does not make any significant difference.

Conversely, for the dataset with 10 classes, the plot in Figure 4b shows that there is little to no separation between the shapes of different classes. It is because the shapes are much more semantically similar, e.g., the shape of Car is similar to a certain kind of vessel, making classification a much more challenging task. In this case, classification accuracy for the contrastive AutoEncoder is better than other methods. It shows that contrastive learning helps separate the embeddings of similar classes, which then helps in classification, as shown in Figure 6a.

Table 3 shows the qualitative results of our shape completion approach on 20% missing data. During our experiments, we also increased the amount of missing data from 20% to 25% and 30% to evaluate the performance of our approach. However, this has an adverse effect on the performance. It is because contrastive learning aims to learn discriminatory features of the shapes, and with increased amount of missing data, the probability of learning such features becomes low. However, the current approach demonstrates the viability of self-supervised learning for point cloud shape completion and classification and provides an interesting direction both theoretically and practically that can be further explored.

Last but not least, from Table 1, it is evident that the mean Chamfer distance for the naive AutoEncoder is slightly lower than other methods, but the overall classification accuracy for the contrastive AutoEncoder is higher than others. It shows how crucial global feature learning is for downstream tasks.

## 5. Conclusions

In this work, we proposed contrastive learning for point cloud shape completion and classification tasks. The purpose of using contrastive learning in our work is to learn the global features of the point cloud classes. The global features are learned through optimizing triplet loss by performing discrimination in the feature space. Furthermore, we used the Chamfer distance function to learn the local features of the shapes. It allowed us to maintain the symmetry and topology of the predicted shapes. We created a unique combination of Chamfer distance and triplet loss, which enabled us to learn both global and local features of the point clouds. We created two subsets consisting of 4 and 10 classes of the ShapeNetCore dataset for our experiments. Our approach achieves comparable classification accuracy on the 4 classes dataset, whereas it achieves state-of-the-art classification accuracy on the 10 classes dataset. Our results are promising, and as a possible extension, we want to further look into other pretext tasks which can help extract useful global features.

## Figures and Tables

**Table 1:** Quantitative results computed by average Chamfer distance $\left(10^{-4}\right)$ between ground truth and completed shapes with respective methods. The lower the Chamfer distance from the ground truth, the better the completed shape is. The naive AutoEncoder performs better than all of the available methods.

RL-GAN	Naive AE	Contrastive AE
10.67	2.34	4.67

**Table 2:** Classification results on completed shapes with 20% missing data. For 4 classes, the naive AutoEncoder achieves better performance and for 10 classes our approach achieves better performance. The best performance of methods for 4 and 10 classes are highlighted in bold face.

No. of Classes	RL-GAN	Naive AE	Contrastive AE
4	96.12%	**97.34**%	97.31%
10	58%	84.2%	**84.9**%

**Table 3:** Comparison of qualitative results of our shape completion pipeline with other methods on 20% missing object shapes.

Ground Truth	Input	Naive AutoEncoder	RL-GAN	Contrastive AutoEncoder
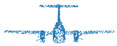	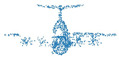	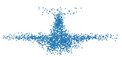	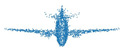	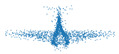
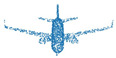	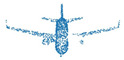	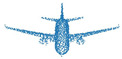	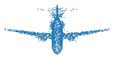	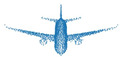
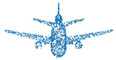	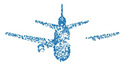	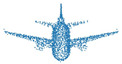	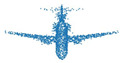	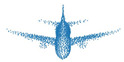
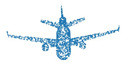	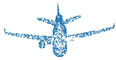	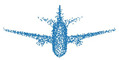	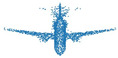	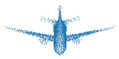
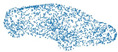	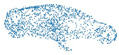	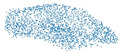	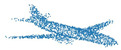	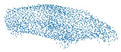
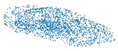	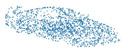	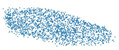	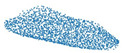	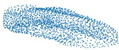
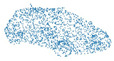	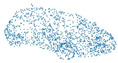	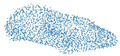	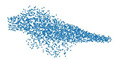	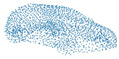
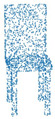	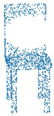	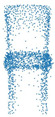	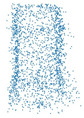	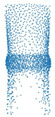
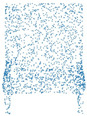	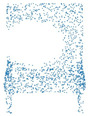	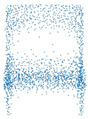	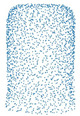	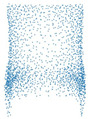
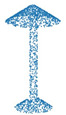	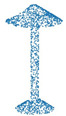	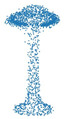	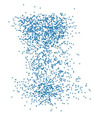	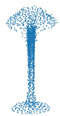
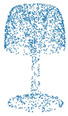	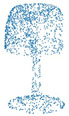	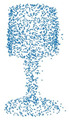	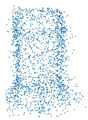	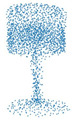
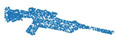	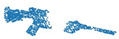	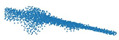	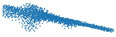	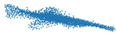
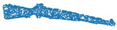	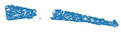	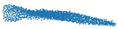	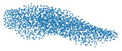	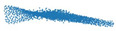
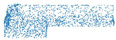	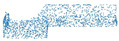	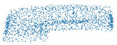	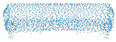	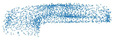
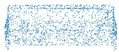	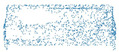	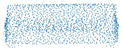	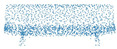	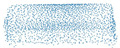
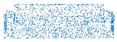	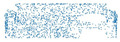	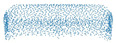	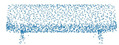	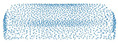
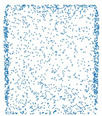	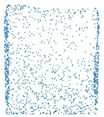	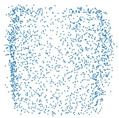	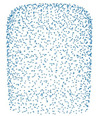	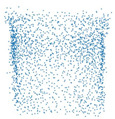
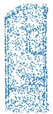	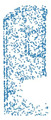	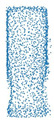	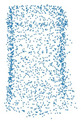	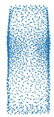
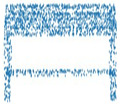	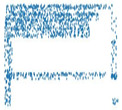	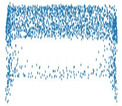	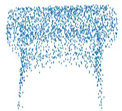	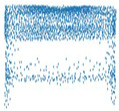
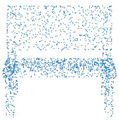	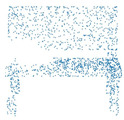	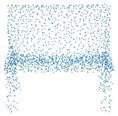	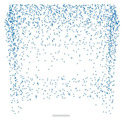	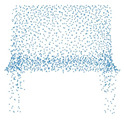
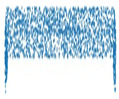	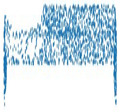	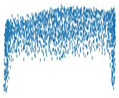	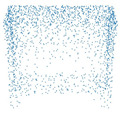	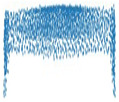
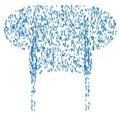	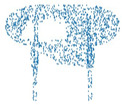	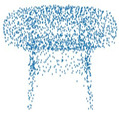	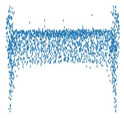	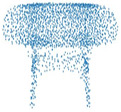
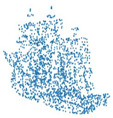	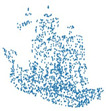	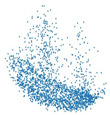	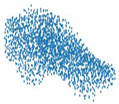	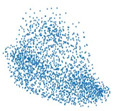
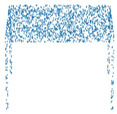	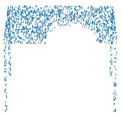	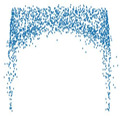	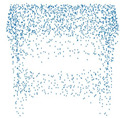	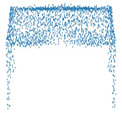
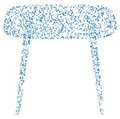	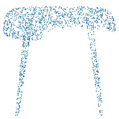	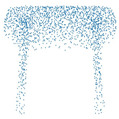	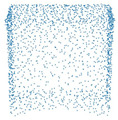	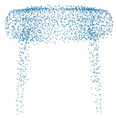
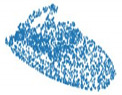	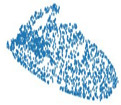	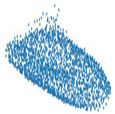	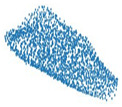	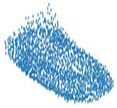

## Data Availability

Not applicable.
